# How and When Does Outcrossing Occur in the Predominantly Selfing Species *Medicago truncatula*?

**DOI:** 10.3389/fpls.2021.619154

**Published:** 2021-02-17

**Authors:** Margaux Jullien, Joëlle Ronfort, Laurène Gay

**Affiliations:** AGAP, INRAE, CIRAD, IRD, Montpellier SupAgro, Université de Montpellier, Montpellier, France

**Keywords:** *Medicago truncatula*, residual outcrossing, predominantly selfing, selfing rate, genetic determinism

## Abstract

Empirical studies on natural populations of *Medicago truncatula* revealed selfing rates higher than 80%, but never up to 100%. Similarly, several studies of predominantly selfing species show variability in the level of residual outcrossing between populations and also between temporal samples of the same population. However, these studies measure global selfing rates at the scale of the population and we do not know whether there is intra-population variation and how outcrossing events are distributed, between genotypes, plants, flowers, or seeds. Theoretical studies predict the maintenance of residual outcrossing in highly selfing species due to environmental (e.g., pollen biology) and/or genetic determinants and decompositions of the variation in outcrossing rate using experimental data can be very informative to test these hypotheses. Here, we focus on one natural population of *M. truncatula* in order to describe precisely its mating system. In particular, we investigated the determinants of the selfing rate by testing for seasonal variations (environmental determinism) and variations between genotypes (genetic determinism). We measured selfing rates in maternal progenies from plants collected widely across a natural population. For each plant, we collected pods from flowers produced at the beginning and at the end of the flowering season to test for a seasonal variation in the outcrossing rate. For each collected offspring, we also estimated the likelihood that it was issued from a self-fertilization event and assessed the genetic component of variation of this mating system measure. We found a significant, albeit small, increase in outcrossing rate in progenies collected at the end [*t*_*m*_ = 0.137 (*SD* = 0.025)] compared to those collected at the beginning [*t*_*m*_ = 0.083 (0.016)] of the flowering season. A significant between genotypes variation in selfing rate was also detected, resulting in a heritability of 9% for the rate of residual outcrossing. Altogether, our work shows that despite a predominantly selfing reproductive mode, *M. truncatula* displays variation in residual outcrossing rate, and that this trait is likely under a complex determinism combining environmental and genetic factors. We discuss the evolutionary implications of our results for the population.

## Introduction

Plant mating systems present a remarkable diversity, which results in a continuous distribution of selfing rates between 0 and 1 over angiosperm species (Igic and Kohn, 2006). Despite widespread hermaphroditism (around 70% of the angiosperms, Richards, 1997), about 50% of angiosperms are obligate outcrossers, and the remainder are either mixed mating (35%, appreciable levels of both outcrossing and selfing) or predominantly selfing (15%, displaying outcrossing rates lower than 10%, Vogler and Kalisz, 2001; Igic and Kohn, 2006). Yet, even in predominantly selfing species, residual outcrossing has been observed in several experimental and natural populations (e.g., Allard and Workman, 1963; Kahler et al., 1975; Bonnin et al., 2001; Bomblies et al., 2010). Actually, complete selfing is seldom encountered in the wild, as shown through a meta-analysis by Winn et al. (2011) in which only 1% of the species reached an estimated outcrossing rate of zero. Little is known about the variability and maintenance of this residual outcrossing and it remains a question of interest. In terms of mechanisms, residual outcrossing is conditioned by the flower’s receptivity to outcross pollen. Lloyd (1992) defined three categories of selfing depending on the timing at which self-fertilization occurs. First, prior selfing, where fertilization between male and female gametes happens in an unopened bud, is mainly affected by the phenology of flower development. The second category is competing selfing, where floral morphology (e.g., herkogamy) can influence the probability of selfing or outcrossing. Finally, delayed selfing, where selfing happens after a waiting time if outcrossing has not occurred, is affected by both flower morphology and pollination environment.

The evolution of plant mating systems is mainly driven by the balance between two opposing forces: the twofold automatic transmission advantage of selfing (Fisher, 1941; Jain, 1976) and inbreeding depression (reviewed in Charlesworth, 2006). Because continued selfing eliminates most of the inbreeding depression by purging deleterious mutations (Lande and Schemske, 1985), reversion to outcrossing is very unlikely once self-fertilization has evolved. This should theoretically allow the fixation of complete selfing (Charlesworth, 1980; Lande and Schemske, 1985; Charlesworth et al., 1990). Yet, mixed−mating regimes, with intermediate and variable selfing rates, are predicted to be evolutionarily stable by models explicitly taking into account ecological mechanisms of pollination, such as pollen limitation (Holsinger, 1991; Cheptou, 2004; Morgan and Wilson, 2005), pollen discounting (Lloyd, 1992; Harder and Wilson, 1998; Johnston et al., 2009; Jordan and Otto, 2012) or a simultaneous increase in the number of selfed and outcrossed ovules (Johnston et al., 2009) (for a review, see Goodwillie et al., 2005). In cleistogamous species, other mechanisms such as adaptive plasticity (Schoen and Lloyd, 1984), variance discounting strategy (Real, 1980; Waller, 1980), avoidance of geitonogamy (Masuda et al., 2001) or of sibling competition (Waller, 1980, 1984), have been hypothesized to maintain a proportion of open flowers (chasmogamous) that can send and receive outcross pollen, along with closed flowers (cleistogamous) that obligatorily self-fertilize (Oakley et al., 2007).

Unlike for mixed-mating regimes, the maintenance of residual outcrossing in predominantly selfing species has rarely been examined. Yet, it could involve different mechanisms, notably because the genetic structure of predominantly selfing species is organized in highly homozygous multilocus genotypes due to the reduced effective recombination (Jullien et al., 2019). First, the variance discounting hypothesis presented above could be reinforced in predominantly selfing populations by the beneficial effects of heterosis and recombination between inbred lines when residual outcrossing is maintained. In a genetic model considering synergistic epistasis between deleterious alleles (i.e., when the effects of mutations alone are smaller than when combined with others), Charlesworth et al. (1991) have shown that an evolutionary stable selfing rate slightly below one could be reached and that complete selfing could be selected against, even under a low magnitude of inbreeding depression. In addition, due to the reduced effective recombination rate in predominantly selfing species (Nordborg, 2000), deleterious mutations are more likely to accumulate through Muller’s ratchet (Muller, 1964). As a consequence, negative disequilibrium can arise, i.e., associations between deleterious and beneficial alleles at different loci (Hartfield, 2016). Kamran-Disfani and Agrawal (2014) used simulations to show that negative disequilibrium is rapidly reduced when the selfing rate is below one. Then, when the selfing rate is allowed to evolve, low levels of outcrossing can be maintained by selection. Furthermore, in completely selfing populations we expect low genetic diversity (Charlesworth and Charlesworth, 1995; Clo et al., 2019), structured into highly differentiated homozygous multilocus genotypes (thereafter called “MLGs”). Residual outcrossing creates recombinant genotypes with new allele combinations, thereby potentially allowing the association of favorable alleles that were otherwise confined into different genetic backgrounds (or homozygous lines). Some outcrossing is therefore beneficial to break apart selection interference between mutations (Hartfield and Glémin, 2016). Unlike for the variance discounting hypothesis in cleistogamous species (Waller, 1984), the beneficial effects of outcrossing described here do not require unpredictable environmental changes. On the contrary, rapidly changing environments can favor the conservation of low levels of residual outcrossing in selfing populations allowing them to rapidly adapt (David et al., 1993; Clo et al., 2020). This parallels the suggestion that the release of genetic variation via sexual reproduction allows adaptation to environmental change and favors the maintenance of sex (Maynard Smith, 1978; Kondrashov, 1993).

Besides its advantage in terms of increased recombination, residual outcrossing could also be maintained by ecological factors related to pollination biology. Pollen limitation can reinforce the reproductive assurance advantage because selfing avoids the occurrence of unfertilized seeds when outcross pollen is limited. Indeed, Larson and Barrett (2000) found that self-compatibility and autogamy were associated with reduced pollen limitation. Using a genetic model, Porcher and Lande (2005) have shown that when the selfing rate is very high, the combined effect of pollen discounting and pollen limitation generates selection that prevents further increase of the selfing rate. This suggests that residual outcrossing could be a best-of-both-worlds strategy.

Finally, the above-mentioned genetic models of mating system evolution all assume a simple genetic determinism of selfing (a single modifier locus) and rarely consider the time to fixation of the selfing allele. Yet, once a sufficiently large selfing rate has been reached, the fitness advantages of increasing the selfing rate might become negligible, which would result in a weaker selection gradient. Moreover, the effective population size is expected to be extremely small at high selfing rates (Glémin, 2007), which could further decrease the efficiency of selection (Burgarella and Glémin, 2017). In light of these arguments, residual outcrossing could just be a prolonged transient state toward complete selfing.

Addressing these different hypotheses to explain the maintenance of residual outcrossing requires further knowledge on the extent of variation in mating regime among populations, and its determinism. The variability of the mating regime at the infra-species scale (among populations, through time, or among individuals) is rarely considered, but was found to be substantial anytime it has been measured (see Figure 1 in Whitehead et al., 2018), even among predominantly selfing species. If there is variability within individual plants, it could reflect a plastic response to specific environmental conditions (e.g., light, temperature, resource availability, etc.) where selfing or outcrossing is favored (adaptive plasticity). High plant density, for example, is expected to increase the opportunities for outcrossing in a population (Karron et al., 1995). Pollinator foraging behavior is another environmental factor that has been shown to generate within plant variation in outcrossing rate (Barrett et al., 1994; Karron and Mitchell, 2012). Alternatively, variability among genotypes would suggest that residual outcrossing is maintained by some genetic factors, i.e., through heritable floral morphology or phenology. Studies looking for the genetic bases of floral evolution have identified several QTLs associated with floral traits that are involved in the selfing syndrome (reviewed in Sicard and Lenhard, 2011). At the intraspecific scale, high heritability values have been found for floral morphology traits (e.g., Campbell, 1996; Ashman and Majetic, 2006). Furthermore, it was shown that genetic variation in floral morphology, such as herkogamy, the spatial separation of style and stigma, was correlated with outcrossing rate variations in populations of several mixed mating species (Karron et al., 1997; Herlihy and Eckert, 2007). The selfing rate of a population is thus most likely determined by complex interactions between genetic and environmental factors and we can expect within population variation, even in the case of predominant selfing.

**FIGURE 1:**
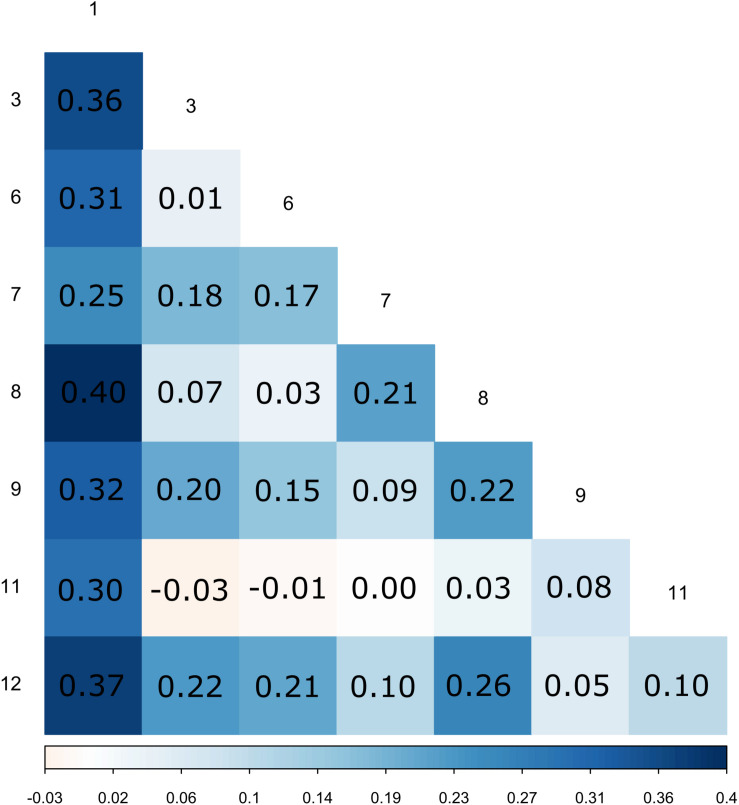
Pairwise *F*_*ST*_ between patches.

### Aims of the Study

The different hypotheses proposed to explain the maintenance of residual outcrossing are difficult to test empirically. Yet, data on variability, spatial but also temporal, can be insightful to understand the mechanisms involved. Here, we investigate in detail the variability in the mating regime of a natural population of the predominantly selfing species *Medicago truncatula* Gaertn (Fabaceae) by focussing on the level of residual outcrossing at a scale that is pertinent for evolution: among individuals within population. *M. truncatula* is an annual plant found in open areas around the Mediterranean Sea. Flowering in *M. truncatula* is undetermined (Moreau et al., 2007), as plants keep producing flowers as long as they have enough resources. The number of flowers produced by a plant is therefore highly variable, and each flower contains between one and 10 ovules (Bataillon and Ronfort, 2006). Although highly selfing, it displays the explosive tripping mechanism (Small, 2011), a floral characteristic shared among the whole *Medicago* genus. When a pollinator visits the flower, the stigma and anthers are projected on the insect’s body, thus coating it with pollen and allowing the stigma to gather pollen from other flowers. Yet, self-fertilization is thought to generally occur within the flower bud, before flower opening (referred to as prior selfing, Lloyd and Schoen, 1992). Nevertheless, complete selfing is rarely observed at the population level. Selfing rates estimated from allozyme data have been reported to range from 0.65 to 1, with a mean selfing rate of 0.96 (Bataillon and Ronfort, 2006). Estimates based on microsatellite data are also strongly skewed toward high rates of selfing and vary between 0.95 and 0.98 (Bonnin et al., 2001; Siol et al., 2008; Jullien et al., 2019). Yet, the maintenance of residual outcrossing in *M. truncatula*, and more generally in predominantly selfing species, is not well understood and knowledge about its variability and determinism is required. The phenology of flower development depends on the accumulation of degree days (Chuine, 2000) and therefore, flowers produced early in the season tend to remain in a closed bud state longer than flowers produced later in the season (L. Gay, field observation). Later flowers are thus more likely to receive outcross pollen. Moreover, flowering is continuous in *M. truncatula*, so that a plant keeps producing flowers while its first pods are maturing, resulting in competition for resources between flowers and pods. This could result in a reduced production of self-pollen (or of lower quality) later in the flowering season and thus in an increase in the likelihood for a plant to be fertilized by outcross pollen received from plants at an earlier phenological stage. The goals of the present study were thus to determine whether residual outcrossing in *M. truncatula* varies during the flowering season and to assess the relative importance of genetic or environmental effects such as plant senescence on the rate of residual outcrossing in a population.

## Materials and Methods

### Study Population and Sampling

The population studied (FR3) is a large and stable population located near Narbonne, in southern France (Aude). It comprises several thousands of individuals. Previous studies have shown that FR3 harbors substantial genetic diversity (samplings in 2004, 2010, and 2014, Jullien et al., 2019) and a strong spatial structure (unpublished results). During spring 2010, the population was monitored and 221 mother plants were randomly chosen in 22 quadrats randomly located in eight different areas (hereafter denominated patches) of the population (Supplementary Figure S1, GPS coordinates provided in Supplementary Table S1). For each mother plant, two pods were collected: one early in the season, often the pod formed by the first flower in May, thereafter called “E” for early; and one toward the end of the season, among the last flowers produced in late June, thereafter called “L” for late. Between one and eight seeds were gathered from each of these pods. The following autumn, the collected seeds were scarified to ease germination and were transferred in petri dishes with water at room temperature for 6 h in the lab. Seeds were then placed in a vernalization chamber at 5°C during 7 days and the germination success was recorded. Seedlings were finally transferred to a greenhouse and leaf material was collected for DNA extraction and for genetic analyses using 20 microsatellite loci. In order to check for differences in seed production or germination rate between early and late pods, a *t*-test of comparison of means was performed using the R software.

### Microsatellite Genotyping

As a first step, equal proportions of leaf material from each of the seedlings collected in a single pod (between 1 and 5) were pooled together to extract DNA. Whenever the number of seedlings per pod exceeded five, the seedlings were split in two pools. DNA bulks were then genotyped for a set of 10 microsatellite loci. For any bulk in which at least one locus displayed two alleles or more, we reiterated the leaf sampling on each individual of the pool and extracted DNA individually. In a second step, the homozygous bulks and the individuals from heterozygous bulks were genotyped on the full set of 20 microsatellites markers (same set as in Jullien et al., 2019), that have been described previously (Baquerizo-Audiot et al., 2001; Arrighi et al., 2006; Ronfort et al., 2006; Siol et al., 2007).

DNA was extracted from 200 mg of frozen leaves. Amplification reactions were performed as described in Siol et al. (2007). Diluted amplification products were analyzed on an ABI prism 3100 Genetic Analyser and results were read using Genemapper 2.5 (Applied Biosystems, Foster City, United States). Individuals and loci with more than 10% missing data were filtered out, resulting in a dataset of 1,729 progenies among which 826 came from early pods and 903 from late pods, genotyped at 19 microsatellite loci.

### Population Genetic Structure

In order to characterize the genetic diversity and spatial genetic structure of the maternal population, we subsampled the filtered dataset to randomly keep a single seed per maternal plant, which resulted in a dataset of 221 individuals. To describe genetic diversity, we computed Nei’s gene diversity (*H*_E_), the observed heterozygosity (*H*_O_), the number of alleles per locus (*n*_A_), and the inbreeding coefficient (*F*_IS_) with the R package hierfstat (Goudet, 2005). The inbreeding coefficient was used to compute an estimate of the selfing rate according to the equation: *s* = $\frac{2F_{IS}}{\left(1+F_{IS}\right)}$, assuming Wright’s inbreeding equilibrium (Wright and Cockerham, 1985). We assessed the within population spatial structure by computing pairwise *F*_*ST*_ between patches according to Weir and Cockerham (1984) with the package hierfstat. In order to account for the large genetic diversity per locus, genetic differentiation between patches was also assessed through Hedrick’s (2005) $G_{ST}^{\prime }$ using the mmod R package (Winter, 2012). Isolation by distance was assessed using both *F*_*ST*_ and $G_{ST}^{\prime }$ estimates of genetic differentiation through Mantel tests using the R package adegenet (Jombart, 2008). Individuals were grouped in multilocus genotypes (MLGs) based on their allelic composition using the R package poppr (Kamvar et al., 2014). An arbitrary threshold error rate corresponding to one genotyping error on one locus was tolerated while assigning individuals to a MLG. Minimum spanning networks based on the genetic distance between MLGs (computed as the proportion of different alleles) were also computed using poppr.

### Selfing Rate Variation Over the Flowering Season: Progeny Array Analyses

The following analyses were performed on the full dataset of 1,729 seeds, for which we specified the identity of the mother plant and the pod (E or L) from which each seed originated. We used the MLTR software, version 3.2 (Ritland, 2002) to compute maximum likelihood estimates of single (*t*_*s*_) and multilocus (*t*_*m*_) outcrossing rates. The difference between the two parameters (*t*_*m*_–*t*_*s*_) provides an estimation of the contribution of biparental inbreeding, or mating between relatives, to the selfing rate. Indeed, if homozygosity at a single locus is compatible with apparent selfing it can also be due to biparental inbreeding. Multilocus genotypes are thus more powerful to tell apart biparental inbreeding and selfing. However, outcrossing between individuals carrying an identical genotype at the 19 SSR loci (which can occur frequently in predominantly selfing populations, Jullien et al., 2019) cannot be detected and the (multilocus) estimated selfing rates can still be overestimated. The correlation of selfing within progeny arrays (*r*_*s*_), which measures the normalized variance in outcrossing rates among families, was also estimated. The estimations were conducted using the Newton-Raphton likelihood optimization algorithm and 1,000 bootstraps with whole family resampling were performed to compute standard errors for the mating system estimates. Allele frequencies were assumed equal between the pollen cloud and the ovule pool. We reiterated the analyses to check for convergence of the model.

In order to test for an effect of flowering time, we first estimated a global outcrossing rate by constraining it to be equal for early and late progenies (i.e., after pooling together progenies from early and late pods). We then reiterated the estimation separately for the progenies from early and late pods. A likelihood ratio test (LRT) was performed to test whether outcrossing rates were significantly different between early and late progenies using ${△}_{dev}=2\left(\sum_{I=1}^{N_{group}}lnLik_{i}-lnLik_{constrained}\right)$. Δ_*dev*_ follows a χ^2^ distribution with *N*_*group*_-1 degrees of freedom, where *N*_*group*_ is the number of subdivisions of the data (here two groups: Early/Late), *lnLik*_I_ is the log-likelihood of the model with independent outcrossing rate for each group and *lnLik*_*constrained*_ is the log-likelihood of the model constrained to estimate a single outcrossing rate. Comparison between outcrossing rates of early vs. late pods were also performed using the Bayesian estimation software BORICE (Koelling et al., 2012) using a chain of 300,000 steps with a burn in period of 15,000 steps on two datasets: offspring from early pods and offspring from late pods. The outcrossing rate tuning parameter, allele frequency tuning parameter and initial population outcrossing rate were set as 0.01, 0.1, and 0.1, respectively. For each dataset, the runs were replicated after changing the starting parameters in order to check for convergence.

### Genetic Variation of Residual Outcrossing

We used the software COLONY version 2.0.6.4 (Jones and Wang, 2010) to perform further parentage analyses on the dataset of 1,729 seeds and estimate an individual probability to stem from a self-fertilization event (*P*_*self*_). Because the true maternal genotypes are unknown in our dataset, COLONY first reconstructs them based on the genotypes of each progeny array. The full likelihood method was used as it is the most accurate method available in COLONY (Wang, 2012). Allele frequencies were calculated from the data without updating by accounting for the inferred relationships.

Taking advantage of the organization in repeated multilocus genotypes in the maternal population, we tested for an effect of the maternal genotype on *P*_*self*_ using a generalized linear mixed model (GLMM), assuming a binomial distribution function and a *logit*-link function, with the R package MCMCglmm (Hadfield, 2010). Pod type (Early or Late) was considered as a fixed effect, whereas the maternal genotype was a random effect. We controlled for three non-genetic random effects: a maternal effect using maternal identity, a pod effect using pod identity, and a spatial structure effect, using patch identity. The model was run with the default weakly informative priors in MCMCglmm (Hadfield, 2010), using 50 million iterations, a burn-in period of 10,000 and a thinning interval of 5,000 to minimize the autocorrelation between samples and ensure a reasonable effective sample size for each effect. Model convergence and mixing were verified by visual examination of posterior traces and autocorrelation values. Heritability was estimated from variance components using the method described by Villemereuil et al. (2016) using the R package QGglmm. Briefly, the variance estimates from a generalized linear mixed model are on a statistically convenient latent scale, and QGglmm converts them to estimate heritability on the scale on which traits are expressed using the classical expression: $h^{2}=\frac{V_{A,obs}}{V_{P,obs}}$ where *V_A__,obs_* and *V*_*P,obs*_ are the additive and phenotypic variance on the observed data scale. In order to test for a potential effect of outcrossing on fitness traits, best linear unbiased predictors (BLUPs) for the maternal genotypes were extracted from the posterior distributions and compared with the mean number of seeds per pod per maternal genotype and the mean germination rate per pod per maternal genotype.

## Results

### Genetic Diversity Analyses

**FIGURE 2:**
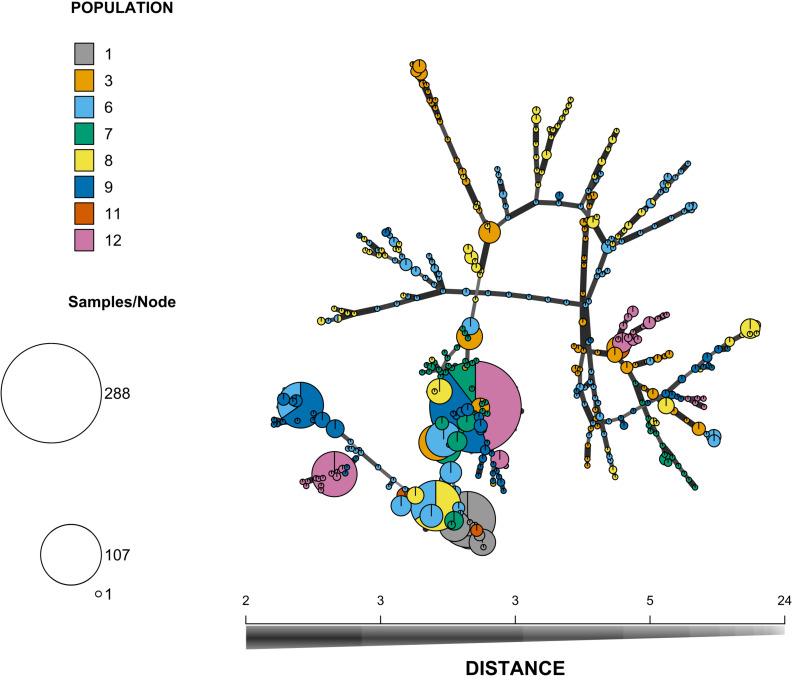
Minimum spanning network of the offspring multilocus genotypes. Each circle represents a MLG and the diameter of the circle represents the frequency of the MLG in the overall sample. Each color represents a patch so that multi-colored circles represent MLGs found in several patches. The length and thickness of the lines linking the MLGs represent their genetic distance computed as the number of different alleles.

All the surveyed microsatellite loci were polymorphic and the number of alleles per locus ranged from 2 to 17, with a mean number *n*_A_ = 7.0 per locus. The mean genetic diversity as measured by *H*_E_ was 0.49 and ranged from 0.31 to 0.83 among patches. The global *F*_IS_ value was 0.90 and ranged from 0.83 to 1 among patches (for details per patch, see Supplementary Table S2), which corresponds to a mean selfing rate of 0.95 for the maternal population. Pairwise *F*_*ST*_ between patches were variable, their value ranging from -0.01 to 0.41 (Figure 1 and see Supplementary Figure S1 for a map of the patches). Mantel tests showed a pattern of isolation by distance between patches (Supplementary Figure S2). Similar results were obtained using $G_{ST}^{\prime }$ measure of genetic differentiation and are reported in Supplementary Figure S3. Using the dataset reduced to a single individual per mother plant (*n* = 221), we were able to cluster the 221 mother plants into 120 MLGs, among which 19% were repeated (represented by more than one individual). The most frequent MLG represented 14% of all the studied mother plants. The minimum spanning network shows the spatial distribution of MLGs across the different patches, as well as the distances among MLGs (Figure 2). It confirms the strong population genetic structure highlighted by the large *F*_*ST*_ values between certain patches (Figure 1) and the AMOVA: the repetitions of a given MLG are clustered together in space and only one MLG (the most frequent one) is observed in three different patches. The other MLGs were found either in a single patch or in only two different patches. Some patches are composed of several low frequency MLGs differing by a few loci (Figure 2).

### Outcrossing Rate Variation Over the Flowering Season

The outcrossing rate estimated on the whole progeny data was around 10% (see multilocus estimates *t*_*m*_ in Table 1). All the reiterations of the analyses gave similar results, which confirmed that the model had reached convergence. The likelihood ratio test (LRT) showed a significantly higher outcrossing rate in late flowers compared to early flowers (Δ_*dev*_ = 62.766; *p* = 2.3 × 10^–15^, Table 1). Using the BORICE software, we also estimated a larger rate of residual outcrossing late in the season (posterior’s median: 0.10; credibility interval 0.08–0.15) than early in the season (posterior’s median: 0.07; credibility interval 0.06–0.11), which also suggests an increase of residual outcrossing with flowering time, even though the posterior distributions overlap slightly (Supplementary Figure S4). The higher outcrossing rate later in the flowering season had no effect on seed production or germination rate as we detected no difference between early and late pods on these two traits (*p* = 0.127 and 0.629, respectively, Supplementary Figures S5, S6).

**TABLE 1:** MLTR estimates of outcrossing rates and results of the likelihood ratio test.

Data	logLik(tm)	*t*_m_ (SD)	*t*_s_ (SD)	*t_m_–t_s_*	*r*_s_
Total	−6,077,986	0.093 (0.021)	0.025 (0.005)	0.068 (0.018)	0.302 (0.044)
Early	−2,666,568	0.083 (0.016)	0.019 (0.004)	0.064 (0.014)	0.368 (0.075)
Late	−3,380,035	0.137 (0.025)	0.031 (0.006)	0.106 (0.020)	0.485 (0.065)

*t_m_ is the multilocus outcrossing rate; t_s_ is the single locus outcrossing rate; r_s_ is the correlation of outcrossing within families. Bracketed values show standard deviations.*

MLTR estimates also provide some insights on how outcrossing events are distributed between plants. The single locus estimates of the outcrossing rate were slightly lower than the multilocus estimates (Table 1), indicating that moderate biparental inbreeding occurs, i.e., that there are outcrossing events between related individuals. Moreover, we estimated a non-null correlation of outcrossing within families (*r*_*s*_, Table 1), suggesting that outcrossing events are not randomly distributed among mother plants.

### Genetic Determinism of Residual Outcrossing

**FIGURE 3:**
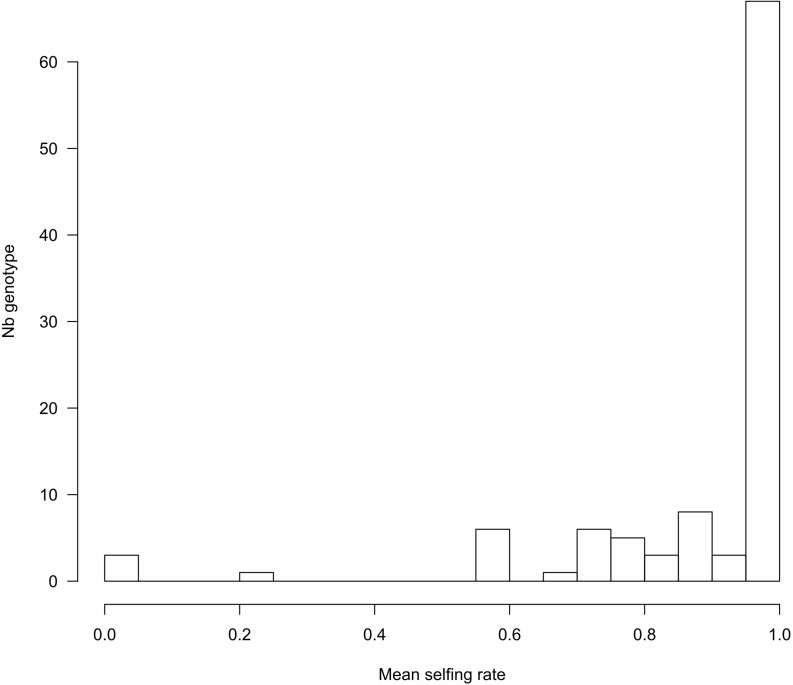
Distribution of selfing rates per maternal genotype (*n* = 103), computed as the mean of *P*_*self*_ from the progeny of each genotype.

Using the 221 progeny arrays, COLONY inferred 103 maternal genotypes, confirming that several mother plants share the same MLG. Note that COLONY infers less genotypes compared to the above analysis (120 MLGs) in which we randomly sampled one seedling per maternal progeny. These two analyses actually deal with two different generations and the sample of one seed per mother plant can overestimate the MLG diversity if it contains outcrossed seeds that will display a unique and new genotype. Yet, both analyses provide very similar results and details about the genetic diversity of the maternal population based on the maternal genotypes inferred by COLONY can be found in Supplementary Table S3 and Supplementary Figure S7. In accordance with MLTR, COLONY estimated a global selfing rate of 0.92 (95% CI: 0.91–0.94). For each progeny, the probability of being selfed (*P*_*self*_) was either 0 or 1, except for two progenies for which *P*_*self*_ = 0.93 and which we converted to 1 in order to obtain binary data to perform the GLMM analysis. The distribution of the average *P*_*self*_ per maternal genotype shows that, in accordance with the high selfing rates estimated, most of the offspring are the result of a selfing event (1,605 selfed vs. 124 outcrossed seeds). However, we found between family variation in the outcrossing rate. Although most of the reconstructed maternal genotypes exhibited selfing rates higher than 0.6 (with 62 genotypes displaying exclusively selfed offspring, Figure 3), in three instances, mother plants produced completely outcrossed progeny.

To determine whether there is an effect of the genotype of the mother on the probability of being selfed (*P*_*self*_) we performed a GLMM analysis. We checked that there was no autocorrelation between samplings of the MCMC chains and that the effective sample sizes for each effect satisfyingly high (>9,000, Table 2). We found a non-significant decrease in the probability of being selfed for seeds produced later in the flowering season (Table 2). No significant effects of the patch of origin or of the maternal plant were detected. On the contrary, significant pod and genotype effects were detected (Table 2), indicating that outcrossing events are not randomly distributed among flowers, nor among maternal genotypes. Using the R package QGglmm, we estimated a mean observed probability of being selfed of 0.906. The observed variance was 0.085, among which 0.008 was genetic variance. This resulted in a broad-sense heritability of 9% for the probability of being selfed. The BLUPs of the probability of being selfed for each maternal genotypes were not correlated with the number of seeds produced (*R*^2^ = 0.009, *p* = 0.76) or with the germination rate (*R*^2^ = 0.01, *p* = 0.94; Supplementary Figures S8, S9).

**TABLE 2:** Results of the generalized linear mixed model performed with MCMCglmm on *P*_*self*_.

Effect	Type	Effective sample size	Posterior mean of the fixed effect (95% CI)	Posterior mean of the variance of random effects (95% CI)
Intercept	Fixed	9,060	6.371 [4.926–7.881]	–
Late	Fixed	9,998	−0.610 [−1.713 to 0.492]	–
Patch	Random	9,998	–	0.706 [9 × 10^–9^–2.4]
Pod	Random	9,348	**–**	**7.678 [3.303–12.42]**
Mother identity	Random	9,599	–	2.053 [5 × 10^–8^–5.947]
Mother genotype	Random	9,221	**–**	**6.003 [1.581–10.85]**

*Effects significantly different from zero are in bold. Posterior means are given on the logit scale.*

## Discussion

In this study, we confirm that, despite its autogamous mating strategy (sensus Richards, 1997, i.e., self-pollination occurs within flower), prior selfing is not systematic in *M. truncatula* flowers and occasional outcrossing occurs. Interestingly, we detected a higher rate of outcrossing in later flowers in accordance with our hypothesis that the end of the flowering season offers more opportunities for outcrossing. Finally, we also detected significant differences in selfing rate between genotypes, which raises the question of the evolution of the mating system in this population.

### Residual Outcrossing and Population Genetic Diversity

The natural population of *M. truncatula* we investigated here presents substantial levels of genetic diversity both at the single and multilocus scale. The most frequent genotype was present in three different patches but only represented 14% of the plants sampled. This frequency is relatively low compared to other *M. truncatula* natural populations (Jullien et al., 2019), which highlights the high level of multilocus diversity encountered in this particular population. Multilocus diversity can directly reflect high levels of single locus diversity or can be generated by residual outcrossing. Indeed, the level of residual outcrossing measured in this population (∼10%) is large compared to values usually found in *M. truncatula* populations (Bonnin et al., 2001; Siol et al., 2007, 2008; Jullien et al., 2019).

As previously reported in other populations of *M. truncatula* (Bonnin et al., 1996, 2001; Siol et al., 2008), this population is strongly spatially structured over a small spatial scale (Supplementary Figure S1) and most repeated MLGs are aggregated in a single patch. This strong aggregation of MLGs in space (even within patches of less than 100 m^2^) could lead to an under-estimation of residual outcrossing because an outcrossing event between individuals carrying the same MLG cannot be detected. In addition, the comparison of multi- and single locus estimates of selfing rates revealed some biparental inbreeding, contrary to what was found by Siol et al. (2008) in another *M. truncatula* population. This biparental inbreeding may occur between MLGs differing at a low number of loci, or within families of related MLGs that could be aggregated in space.

### Seasonal Variation of the Residual Outcrossing Rate

In accordance with our primary hypothesis, we detected a significant increase of the population outcrossing rate along the flowering season, with pods produced early in the flowering season displaying a lower outcrossing rate than pods produced by the same mother plant at the end of the flowering season. The differences observed between MLTR and BORICE estimates are probably due to the fact that MLTR is more suited to the analysis of families with a large number of offspring than BORICE (Koelling et al., 2012). An effect in the same direction, though not significant, was detected in the model fitted on the individual probability of being self-fertilized estimated by COLONY, that accounted for flower, maternal, spatial and genetic effects. *P*_*self*_, the probability of being selfed, was estimated by Colony, but the estimation error cannot be propagated in the GLMM. This could explain why the flowering season effect did not appear significant. Similarly, an increase in outcrossing rate between the first and the second flowers of the inflorescences was observed in inflorescences of *Aquilegia buergeriana* var. *oxysepala* (Itagaki et al., 2016). The authors also reported variations in the number of pollen grains produced by those two types of flowers, which is hypothesized to explain the higher selfing rate in the first flowers. In *Lychnis flos-cuculi*, the selfing rate was shown to be higher in early opening flowers because the proportion of outcross pollen in the pollen cloud was lower. Indeed, due to the combination of basipetal blooming and protandry, the early flowering flowers encounter less outcross pollen (Dulya and Mikryukov, 2016). The reverse trend was observed in *Incarvillea sinensis*, where the outcrossing rate decreased along the flowering season, indicating deteriorating conditions for cross-pollination (Yin et al., 2016). Finally, Colicchio et al. (2020) found no effect of the season on the individual selfing rate of the mixed-mating plant *Mimulus guttatus* (Colicchio et al., 2020).

Several hypotheses related to flower traits could explain the variation in residual outcrossing we observed. First, flower development is faster late in the season due to higher daily temperatures. This could speed up the opening of the flowers and provide more opportunities for outcrossing. The quantity of pollen or ovules could also vary between early and late flowers as ecological factors, e.g., reduced pollinator service toward the end of the flowering season, could select for a temporal shift in sex allocation in outbreeding plants, such as increased male investment in late flowers (Brunet and Charlesworth, 1995). Yet, a study measuring the changes in pollen-ovule (P:O) ratios over the flowering season in two pairs of sister taxa in the genus *Clarkia* in a controlled greenhouse experiment showed that, contrary to their outcrossing sister species, selfing species present stable P:O ratios over time (Mazer et al., 2009). However, this result was less clear in natural conditions (Delesalle et al., 2008).

Such increase in the outcrossing rates later in the season resembles “delayed outcrossing” at the plant level, by analogy with delayed selfing which has been extensively described (Goodwillie and Weber, 2018). Delayed selfing is considered as a “best-of-both-worlds” mating system because it combines the advantages of allogamy in terms of recombination and the reproductive assurance of selfing when outcrossing is not possible. Delayed selfing is for example invoked to explain the decreasing outcrossing rate along the flowering season in *Incarvillea sinensis* (Yin et al., 2016). On the contrary, *M. truncatula* is predominantly selfing, but residual outcrossing is observed in all flowers and is slightly more frequent toward the end of the flowering season. Since delayed outcrossing increases recombination, it could be beneficial through the breaking of negative disequilibrium (Hartfield, 2016) or selection interference between advantageous mutations (Hartfield and Glémin, 2016). However, it could also break co-adapted gene pools (Allard et al., 1972, 1993). In our analysis, we did not detect a cost for increased outcrossing, as the number of seeds and the germination rate were not affected. Therefore, “delayed outcrossing” could also be considered as a best-of-both-worlds mating system in *M. truncatula*. This raises the question of the genetic basis underlying the variability in the outcrossing rate as it is a prerequisite for the trait to evolve.

### Genetic Determinism of Residual Outcrossing

Our analysis showed that the between family variation in selfing rate (as measured by MLTR through the correlation of selfing within families, *r*_*s*_) in *M. truncatula* is genetically determined. The genetic component we estimated was however reduced, with around 9% of the variation of the individual probability of being selfed explained by the maternal genotype. Heritability estimates for the selfing rate are extremely scarce in the literature. To our knowledge, a single study on the mixed mating species *Mimulus ringens* found a broad-sense heritability for the outcrossing rate of 0.37 (Karron et al., 1997). Yet, and unlike *M. ringens*, *M. truncatula* is predominantly selfing and the variation range in residual outcrossing is narrow, which made the detection of a genetic determinism even more challenging.

However, our diversity analysis highlights shortcomings of our experimental design. First, if the presence of repeated genotypes in the population enables testing for a genetic determinism of the selfing rate, their strong aggregation over space, with most of the repeated MLGs located in the same patch, can lead to confusion between environmental and genetic effects. Such strong spatial structure is typical for predominantly selfing species, and has already been described in natural populations of *M. truncatula* (Bonnin et al., 1996). Second, only 20% of the mother plants sampled carried a repeated MLG and were useful to test for a genetic determinism of the trait. This could limit our power to partition the variability in selfing rates between genotypic and environmental or maternal effects. A larger experiment where a larger number of mother plants per genotype would be randomized across the population could provide a clearer answer to the question of the genetic determinism of the selfing rate.

Assuming that the genetic variance we detected for residual outcrossing is not too much inflated by environmental effects shared within a genotype, we can address the question of the consequences for the evolution of the mating system in this population. As detailed in the Introduction, there are several advantages to residual outcrossing such as the advantage of recombination or an ecological advantage related to pollination biology. We can therefore expect that some residual outcrossing should be selectively maintained in the population. Yet, the low heritability detected implies that most of the variance for residual outcrossing is environmental and among pods (not at the patch or mother plant level). This suggests that the effect of temporal variability in the flower’s environmental conditions prevails over spatial variability (at the patch or mother plant scale). This means that there is plasticity for residual outcrossing in relation to temporally changing environments like biotic conditions, local climate or resources availability. Such plastic responses of the mating system have already been reported in relation to pollinator behavior (Barrett et al., 1994; Karron et al., 2009; Karron and Mitchell, 2012), or resources availability (Waller, 1980). Given the prevalence of environmental variance on residual outcrossing, we expect a low efficiency of selection and no evolution of the mating system in the population of *M. truncatula* studied here.

## Conclusion

In conclusion, the rate of residual outcrossing in *M. truncatula* appears to be variable during the flowering season, between flowers and between genotypes, although the selfing rate always remains very high. The within population variability of the mating system is seldom assessed even though it is the scale at which evolution occurs. Selfing rates are generally reported at the species level (Igic and Kohn, 2006), yet, it has been shown that there is substantial variability between populations (Whitehead et al., 2018). Here, we provide an example of within population variability of the mating system. This temporal variability in the mating system in *M. truncatula* could explain why this predominantly selfing species maintained the tripping mechanism as an adaptation to pollination. A review showed that observing floral traits that promote pollination specialization and autonomous selfing in the same flower is not paradoxical, and is frequent, but generally occurs in self-compatible species that mostly outcross and use delayed selfing as a reproductive assurance (Fenster and Martén-Rodríguez, 2007). Our study shows that residual outcrossing has a complex determinism combining environmental and genetic effects, with a low yet significant heritability. Studies comparing the development of flowers and their pollen production during the flowering season could be helpful to better understand the temporal variations of outcrossing rate in this *M. truncatula* population.

## Data Availability Statement

The original contributions presented in the study are publicly available. This data can be found here: https://doi.org/10.15454/WPUFUD.

## Author Contributions

LG and JR conceived and performed the research. LG performed the data collection in 2010 and ran primary analyses. MJ performed the data analyses during her Ph.D. and wrote the article with the help of all authors who critically reviewed and approved the text.

## Conflict of Interest

The authors declare that the research was conducted in the absence of any commercial or financial relationships that could be construed as a potential conflict of interest.

## Supplementary Material

Supplementary Figure 1 Map of the FR3 population.

Supplementary Figure 2 Detection of isolation by distance patterns using Weir and Cockerham’s (1984) *F*_*ST*_ measure of genetic differentiation.

Supplementary Figure 3 Spatial structure analyses using Hedrick’s (2005) $G_{ST}^{\prime }$ differentiation measure.

Supplementary Figure 4 Distribution of posterior probabilities of the outcrossing rate estimated with BORICE.

Supplementary Figure 5 Distribution of the number of seeds per pod.

Supplementary Figure 6 Distribution of the germination rate for early or late pods.

Supplementary Figure 7 Minimum spanning network based on the maternal genotypes inferred by COLONY.

Supplementary Figure 8 Best linear unbiased predictors (BLUP) of the maternal genotypes for the number of seeds per pod.

Supplementary Figure 9 Best linear unbiased predictions (BLUP) of the maternal genotypes for the mean germination rate per pod.

Supplementary Table 1 GPS coordinates of the 8 patches from the FR3 *M. truncatula* population.

Supplementary Table 2 Genetic diversity within the experimental patches computed from the progeny genotypes.

Supplementary Table 3 Single and multilocus genetic diversity within the experimental patches of the maternal genotypes as inferred by COLONY.
