# Supplementary material for: How and When Does Outcrossing Occur in the Predominantly Selfing Species Medicago truncatula?
Source: Front Plant Sci. 2021 Feb 17;12:619154. doi: 10.3389/fpls.2021.619154 (PMC7925993; doi:10.3389/fpls.2021.619154)

**Figure S2: Detection of isolation by distance patterns using Weir & Cockerham’s (1984) *F_ST_* measure of genetic differentiation**

**Figure 2.1: Simulated correlations between pairwise *F_ST_* matrix and geographic distance matrix under the absence of spatial structure (permuted values).**

The black dot represents the original value of the correlation between genetic and geographic distances


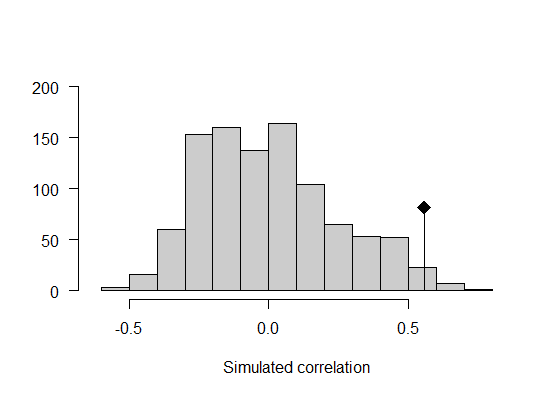


**Figure 2.2: Distribution of pairwise *F_ST_* over distance (in meters)**

The red line is the linear regression of pairwise ***F_ST_*** over geographic distance


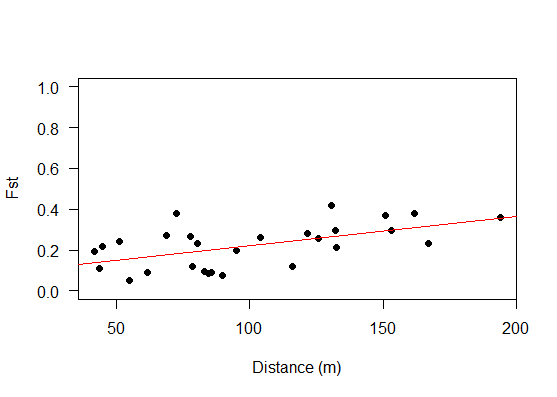

Supplement: Supplementary Figure 1 — Map of the FR3 population. [file Data_Sheet_1.zip › Figure 2.DOCX]
