# Supplementary material for: How and When Does Outcrossing Occur in the Predominantly Selfing Species Medicago truncatula?
Source: Front Plant Sci. 2021 Feb 17;12:619154. doi: 10.3389/fpls.2021.619154 (PMC7925993; doi:10.3389/fpls.2021.619154)

**Figure S3: Spatial structure analyses using Hedrick’s (2005) G’st differentiation measure**

| Patch | 1 | 3 | 6 | 7 | 8 | 9 | 11 |
| --- | --- | --- | --- | --- | --- | --- | --- |
| 3 | 0.682 |  |  |  |  |  |  |
| 6 | 0.562 | 0.194 |  |  |  |  |  |
| 7 | 0.476 | 0.514 | 0.445 |  |  |  |  |
| 8 | 0.654 | 0.223 | 0.129 | 0.470 |  |  |  |
| 9 | 0.578 | 0.556 | 0.440 | 0.205 | 0.527 |  |  |
| 11 | 0.626 | 0.238 | 0.299 | 0.399 | 0.298 | 0.494 |  |
| 12 | 0.615 | 0.528 | 0.484 | 0.175 | 0.507 | 0.147 | 0.448 |

**Table: Pairwise G’st (according to Hedrick, 2005) between patches**

We performed Mantel tests to test for a correlation between pairwise G’st values and geographic distances using the adegenet R package. We performed 100 permutations to simulate the absence of spatial structure and compare with the observed correlation.

**Figure S3.1: Simulated correlations between pairwise Gst matrix and geographic distance matrix under the absence of spatial structure (permuted values).**

The black dot represents the original value of the correlation between genetic and geographic distances


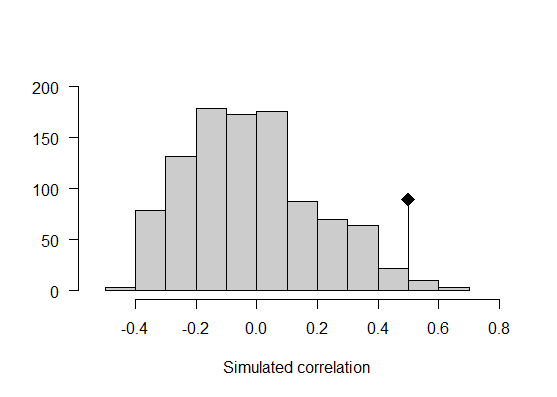


**Figure S3.2: Distribution of pairwise G’st over distance (in meters)**

The red line is the linear regression of pairwise G’st over geographic distance


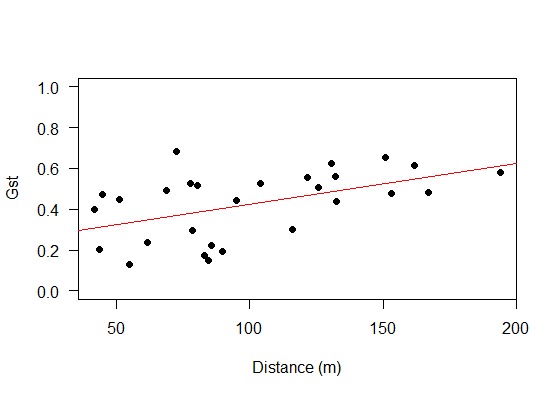

Supplement: Supplementary Figure 1 — Map of the FR3 population. [file Data_Sheet_1.zip › Figure 3.DOCX]
