# Supplementary material for: How and When Does Outcrossing Occur in the Predominantly Selfing Species Medicago truncatula?
Source: Front Plant Sci. 2021 Feb 17;12:619154. doi: 10.3389/fpls.2021.619154 (PMC7925993; doi:10.3389/fpls.2021.619154)
Supplement: Supplementary Figure 1 — Map of the FR3 population. [file Data_Sheet_1.zip › Figure 4.DOCX]

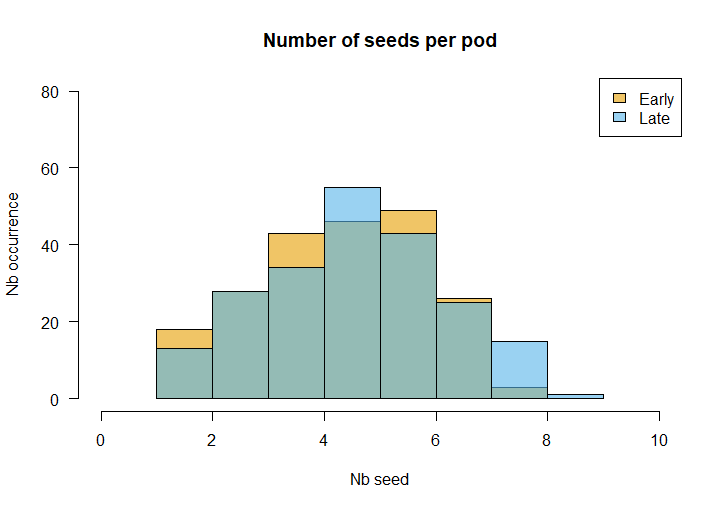


**Figure S5: Distribution of the number of seeds per pod.**

Early pods are represented in orange and late pods in blue. A Pearson’s Chi-squared test revealed no significant difference between the number of seeds in pods produced early in the flowering season and pods produced at the end of the flowering season (*p*-value = 0.127).
