# Supplementary material for: How and When Does Outcrossing Occur in the Predominantly Selfing Species Medicago truncatula?
Source: Front Plant Sci. 2021 Feb 17;12:619154. doi: 10.3389/fpls.2021.619154 (PMC7925993; doi:10.3389/fpls.2021.619154)
Supplement: Supplementary Figure 1 — Map of the FR3 population. [file Data_Sheet_1.zip › Figure 7.DOCX]

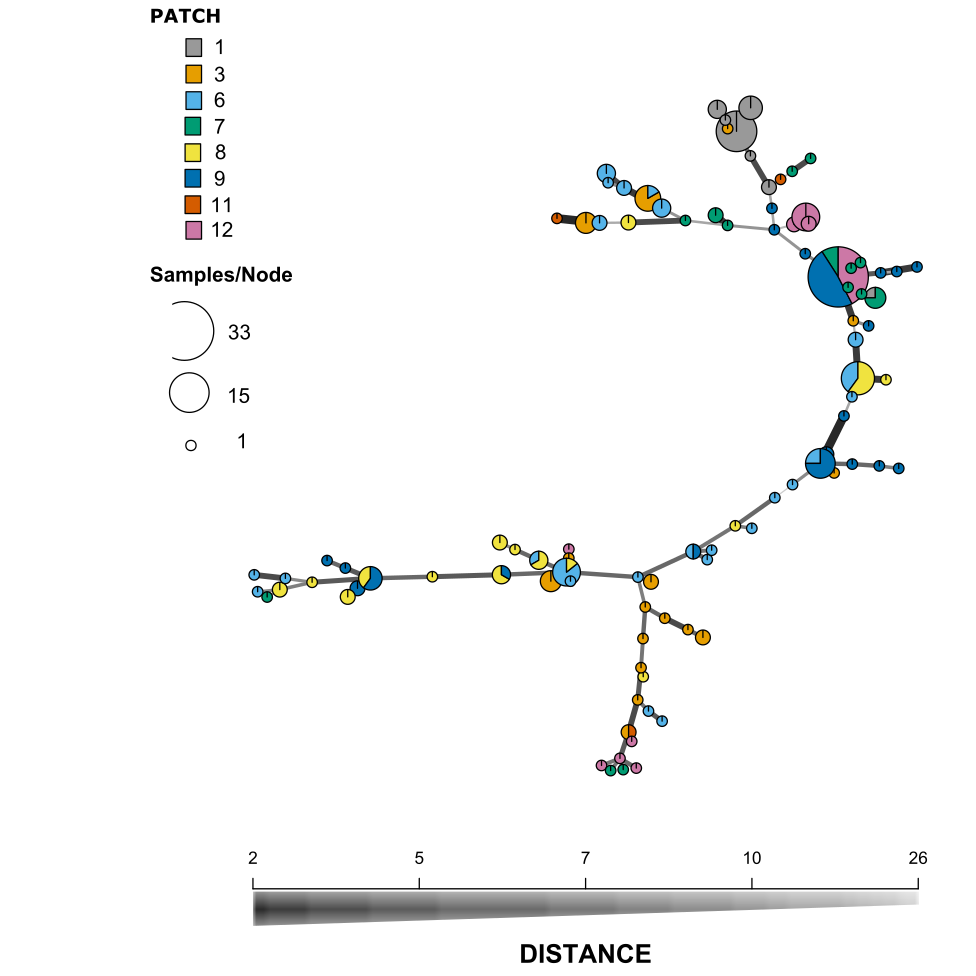


**Figure S7: Minimum spanning network based on the maternal genotypes inferred by Colony.**

Each circle represents a MLG and the diameter of the circle represents the frequency of the MLG in the overall sample. Multi-coloured circles represent MLGs found in several patches. The length and thickness of the lines linking the MLGs represents their genetic distance computed as the number of different alleles.
