# Supplementary material for: How and When Does Outcrossing Occur in the Predominantly Selfing Species Medicago truncatula?
Source: Front Plant Sci. 2021 Feb 17;12:619154. doi: 10.3389/fpls.2021.619154 (PMC7925993; doi:10.3389/fpls.2021.619154)
Supplement: Supplementary Figure 1 — Map of the FR3 population. [file Data_Sheet_1.zip › Table 1.DOCX]

**Table S1: GPS coordinates of the 8 patches of *Medicago truncatula* from the FR3 population (near Narbonne, south of France)**

| Patch | Longitude | Latitude |
| --- | --- | --- |
| 1 | 2.998739 | 43.144257 |
| 3 | 2.999147 | 43.144838 |
| 6 | 3.000237 | 43.144717 |
| 7 | 2.999656 | 43.145459 |
| 8 | 3.000075 | 43.145198 |
| 9 | 2.99972 | 43.145848 |
| 11 | 2.999149 | 43.145392 |
| 12 | 2.998699 | 43.145713 |
