# Supplementary material for: How and When Does Outcrossing Occur in the Predominantly Selfing Species Medicago truncatula?
Source: Front Plant Sci. 2021 Feb 17;12:619154. doi: 10.3389/fpls.2021.619154 (PMC7925993; doi:10.3389/fpls.2021.619154)
Supplement: Supplementary Figure 1 — Map of the FR3 population. [file Data_Sheet_1.zip › Table 2.DOCX]

**Table S2: Genetic diversity within the experimental patches computed from the progeny genotypes.**

*N_plant_* is the number of maternal plants sampled in the patch, *N_seed_* is the number of offspring from the pods collected in each patch; *H_E_* is Nei’s genetic diversity; *H_O_* is the observed heterozygosity; *F_IS_* is the inbreeding coefficient; *nMLG* is the number of MLGs; *singleMLG* is the proportion of unique MLGs; and *MFMLG* is the frequency of the most frequent MLG.

| Patch | *N_plant_* | *H_E_* | *H_O_* | *F_IS_* | *N_seed_* | *nMLG* | *singleMLG* | *MFMLG* |
| --- | --- | --- | --- | --- | --- | --- | --- | --- |
| 1 | 29 | 0.31 | 0.01 | 0.96 | 221 | 13 | 0.01 | 0.48 |
| 3 | 28 | 0.59 | 0.06 | 0.91 | 212 | 64 | 0.19 | 0.18 |
| 6 | 41 | 0.59 | 0.08 | 0.87 | 320 | 114 | 0.26 | 0.14 |
| 7 | 19 | 0.49 | 0.08 | 0.84 | 156 | 55 | 0.28 | 0.19 |
| 8 | 27 | 0.57 | 0.08 | 0.83 | 212 | 77 | 0.26 | 0.27 |
| 9 | 44 | 0.42 | 0.04 | 0.90 | 300 | 60 | 0.11 | 0.42 |
| 11 | 3 | 0.55 | 0.00 | 1.00 | 23 | 3 | 0.00 | 0.39 |
| 12 | 30 | 0.44 | 0.04 | 0.90 | 285 | 39 | 0.08 | 0.46 |
| Total | 221 | 0.49 | 0.05 | 0.90 | 1729 | 415 | 0.16 | 0.17 |
