# Supplementary material for: How and When Does Outcrossing Occur in the Predominantly Selfing Species Medicago truncatula?
Source: Front Plant Sci. 2021 Feb 17;12:619154. doi: 10.3389/fpls.2021.619154 (PMC7925993; doi:10.3389/fpls.2021.619154)
Supplement: Supplementary Figure 1 — Map of the FR3 population. [file Data_Sheet_1.zip › Table 3.DOCX]

**Table S3: Single and multilocus genetic diversity within the experimental patches of the maternal genotypes as inferred by Colony.**

*N_plant_* is the number of maternal plants sampled in the patch; *H_E_* is Nei’s genetic diversity; *H_O_* is the observed heterozygosity; *F_IS_* is the inbreeding coefficient; *nMLG* is the number of MLGs; *singleMLG* is the proportion of unique MLGs; and *MFMLG* is the frequency of the most frequent MLG.

| Patch | *N_plant_* | *H_E_* | *H_O_* | *F_IS_* | *nMLG* | *singleMLG* | *MFMLG* |
| --- | --- | --- | --- | --- | --- | --- | --- |
| 1 | 29 | 0.31 | 0.01 | 0.97 | 8 | 0.14 | 0.52 |
| 3 | 28 | 0.61 | 0.14 | 0.78 | 16 | 0.39 | 0.18 |
| 6 | 41 | 0.61 | 0.17 | 0.72 | 25 | 0.41 | 0.15 |
| 7 | 19 | 0.54 | 0.18 | 0.69 | 14 | 0.58 | 0.16 |
| 8 | 27 | 0.57 | 0.21 | 0.67 | 15 | 0.26 | 0.22 |
| 9 | 44 | 0.52 | 0.13 | 0.75 | 20 | 0.34 | 0.36 |
| 11 | 3 | 0.78 | 0.00 | 1.00 | 3 | 1.00 | 0.33 |
| 12 | 30 | 0.46 | 0.07 | 0.83 | 9 | 0.17 | 0.47 |
| Total | 221 | 0.54 | 0.11 | 0.79 | 98 | 0.29 | 0.15 |
