# Supplementary figures and images for: How and When Does Outcrossing Occur in the Predominantly Selfing Species Medicago truncatula?
Source: Front Plant Sci. 2021 Feb 17;12:619154. doi: 10.3389/fpls.2021.619154 (PMC7925993; doi:10.3389/fpls.2021.619154)

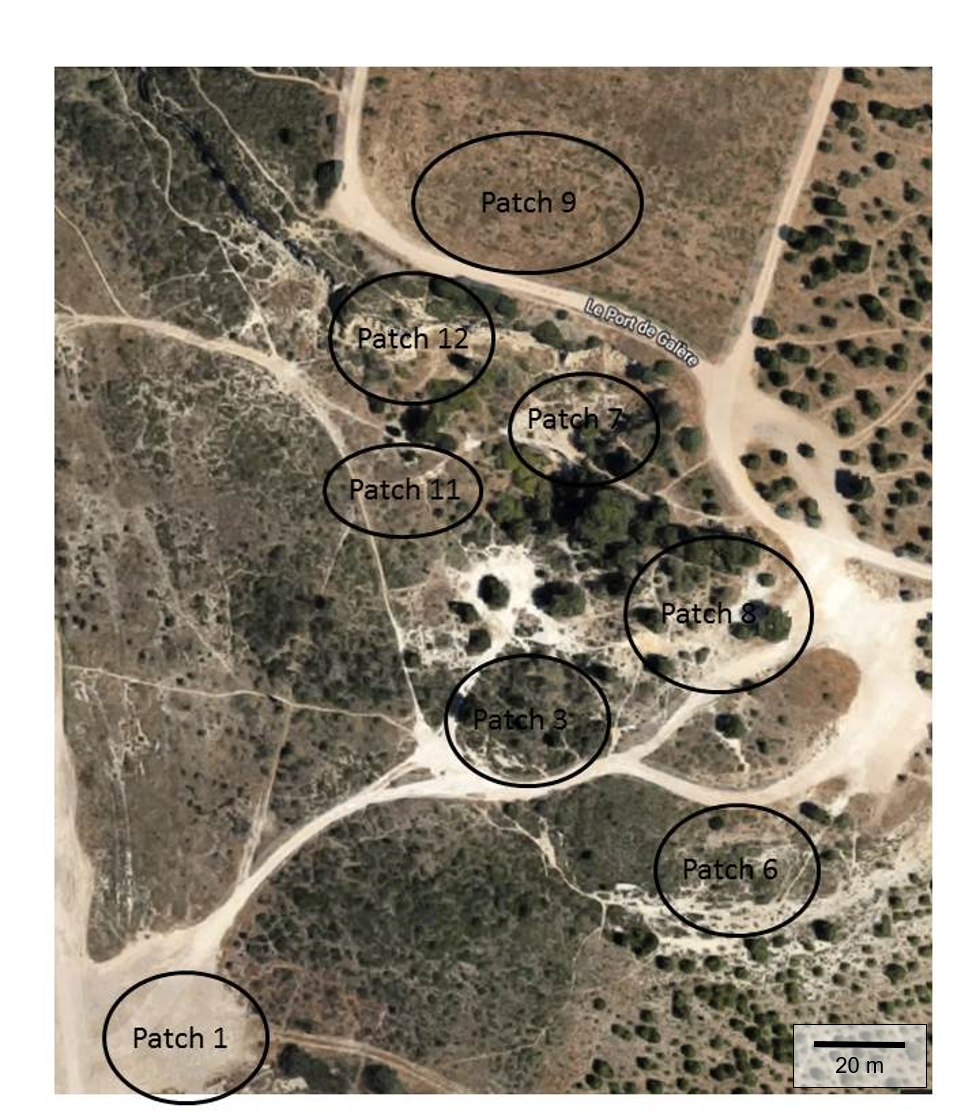


**Figure S1: Map of the FR3 population**

Supplement: Supplementary Figure 1 — Map of the FR3 population. [file Data_Sheet_1.zip › Figure 1.DOCX]
